# Encephalitis in a patient with hypopharynx cancer treated with immune checkpoint inhibitors and radiotherapy: a case report and review of the literature

**DOI:** 10.1007/s00432-023-05328-3

**Published:** 2023-09-07

**Authors:** Yan Kang, Hongchao Zhen, Nina Ma, Hong Zhao, Bangwei Cao

**Affiliations:** 1 Department of Oncology, Beijing Friendship Hospital, Capital Medical University, No. 95 Yong An Road, Xicheng District, Beijing, 100050 People’s Republic of China; 2 Department of Radiotherapy, Beijing Friendship Hospital, Capital Medical University, No. 95 Yong An Road, Xicheng District, Beijing, 100050 People’s Republic of China

**Keywords:** Immune checkpoint inhibitors (ICIs), Neurologic immune-related adverse events (NirAEs), Encephalitis, Camrelizumab, Programmed cell death-1 (PD-1)

## Abstract

Hypopharyngeal cancer (HPC) has one of the most unfavorable prognoses among head and neck squamous cell carcinomas. Immunotherapy in combination with chemotherapy, the same as conventional induction chemotherapy, has emerged as a vital part of the induction therapy protocol for HPC. Meanwhile, the incidence of immune-related adverse events is increasing. In this light, we present the first reported case of immune-associated encephalitis in a patient with hypopharyngeal cancer treated with Camrelizumab (a PD-1 inhibitor). After receiving immunotherapy combined with chemotherapy as induction therapy, along with concurrent chemoradiotherapy, the patient presented with symptoms of fatigue, tremors, drowsiness, and an abnormal signal in the right temporal lobe as shown on a brain magnetic resonance imaging (MRI). Despite the minor elevation in protein and IgG index observed in the lumbar puncture, there is no evidence of abnormal autoantibodies or evidence of pathogenic infection. Following a thorough multidisciplinary consultation, the patient is suspected to be afflicted with immune-related autoimmune encephalitis. Intravenous methylprednisolone was prescribed as an empirical treatment at an initial dosage of 120 mg/day for 3 days, followed by steroid tapering. Finally, the patient experienced complete neurologic and radiographic (brain MRI) recovery. This case serves as a critical reminder that encephalitis is a potential diagnosis that should never be overlooked in patients undergoing immunotherapy who present with abnormal signs of the brain. The timely diagnosis and initiation of immunosuppressive therapy are key components of treating ICI-associated encephalitis.

## Background

The approval of immune checkpoint inhibitors (ICIs) such as cytotoxic T-lymphocyte-associated antigen 4 (CTLA-4) receptor and programmed cell death-1 (PD-1) or its ligand (PD-L1) has led to their critical role in a growing number of clinical treatment strategies. The increasing use of ICIs in clinical practice has resulted in a rise in immune-related adverse events (irAEs). As opposed to irAEs involving other organs, neurological complications of ICIs are rare, with an incidence of 1–14%. However, their potential to significantly impact patients’ quality of life and disrupt cancer treatment underscores their significance(Cuzzubbo et al. 2017; Kao et al. 2017; Larkin et al. 2017; Spain et al. 2017; Dubey et al. 2020; Martínez-Vila et al. 2021). Neurologic irAEs (NirAEs) encompass a range of toxicities affecting the central and peripheral nervous systems, such as myositis, myasthenia gravis, Guillain-Barré syndrome, posterior reversible encephalopathy syndrome, demyelinating polyradiculoneuropathy, aseptic meningitis, and autoimmune encephalitis (Wilgenhof and Neyns 2011; Maur et al. 2012; Bot et al. 2013; Vitt et al. 2018; Mohn et al. 2019; Safa et al. 2019; Nersesjan et al. 2021). Immune-related encephalitis occurs in less than 1% of patients who receive ICIs (Perrinjaquet et al. 2019; Martínez-Vila et al. 2021). However, if not diagnosed and treated promptly, the mortality rate can reach up to 19% (Johnson et al. 2019; Chisaki et al. 2022). We present a case of suspected immune-associated encephalitis in a patient with hypopharynx cancer who received Camrelizumab in People’s Republic of China. To the best of our knowledge, this represents the first documented case of encephalitis associated with immune therapy using Camrelizumab.

## Case presentation

**Fig. 1:**
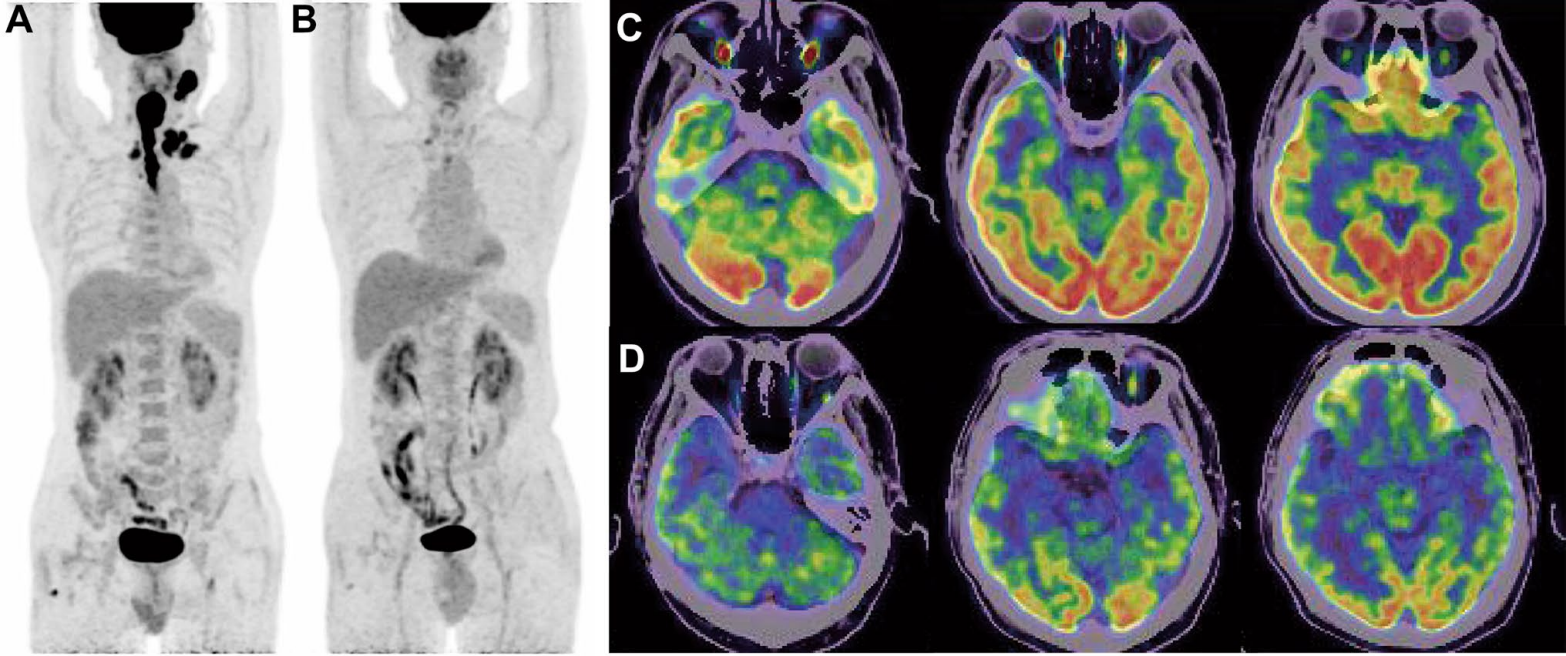
The imaging changes of PET/CT before and after four cycles of Camrelizumab plus albumin paclitaxel combined with cisplatin. **A** Whole-body PET/CT showed that the patient’s primary lesion and metastasis in lymph nodes. **B** Whole-body PET/CT showed the patient achieved PR after four cycles of immunotherapy with chemotherapy. **C** and **D** PET/CT showed that corresponding areas were normal before and after four cycles of immunotherapy with chemotherapy

Written consent was obtained. A 58-year-old Asian man was diagnosed with stage IV hypopharynx cancer in our oncology department in 2021. He had been managing hypertension and type 2 diabetes without medication for 10 years. He had an allergy to sulfonamide medication. In addition, his mother had passed away due to liver cancer. At the time of the primary diagnosis of hypopharynx cancer, he presented with bilateral supraclavicular lymph node metastases and esophageal invasion. However, the positron emission tomography-computed tomography (PET-CT) scan (Fig. 1A, C) revealed no brain metastases or other distant metastases, and the clinical evidence supported this finding. Through multidisciplinary consultation, the experts recommended a sequential therapy approach involving induction chemotherapy followed by concurrent chemoradiation. Based on the patient’s intention and the proven efficacy of immune checkpoint inhibitors (ICIs) in head and neck squamous cell cancer, the treatment plan involved intravenous injection of the PD-1 antibody Camrelizumab (200 mg) along with a standard dose of albumin paclitaxel (260 mg/m^2^) and cisplatin (75 mg/m^2^) every 3 weeks. He experienced grade IV myelosuppression and granulocytopenia fever during treatment. However, the blood test results returned to expected levels after treatment with recombinant human granulocyte colony-stimulating factor. The patient demonstrated a partial remission (PR) after four cycles of immunotherapy in combination with chemotherapy (Fig. 1B).

**Fig. 2:**
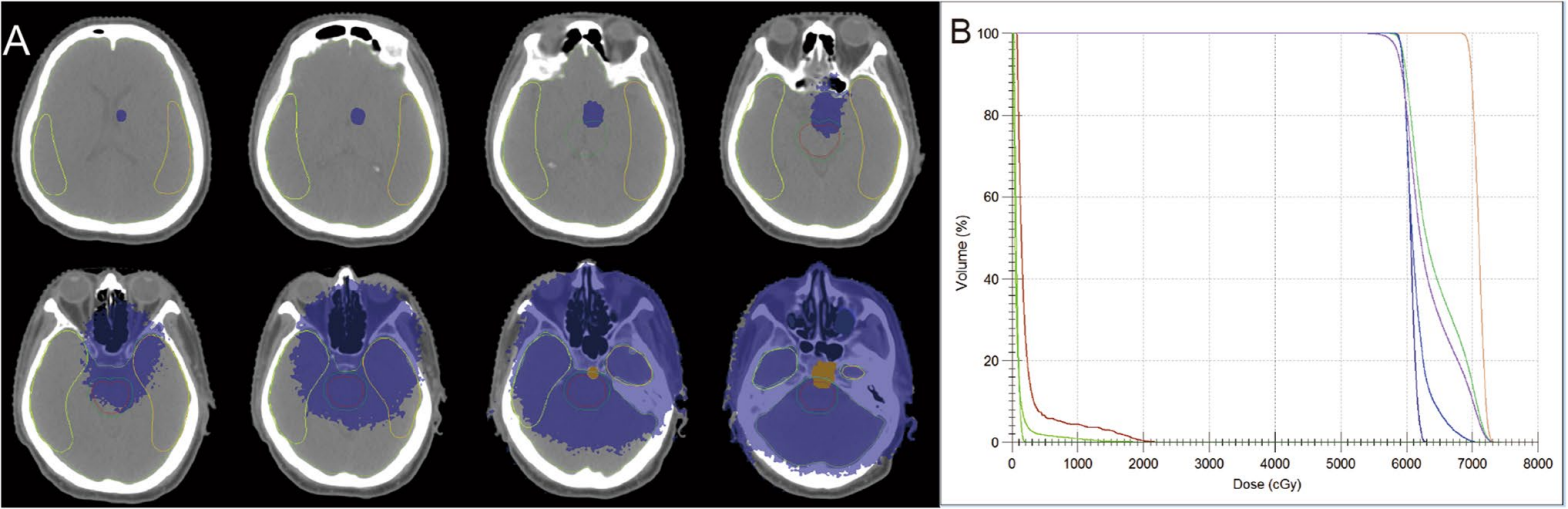
The dose distribution map in the brain and Dose-Volume Histogram. **A** The purple area is the 2 Gy dose distribution region, and the orange area is the 3 Gy dose distribution. The area within the yellow line is the right temporal lobe, the orange line is the left temporal lobe, and the green line indicates the whole brain. **B** The light brown is GTV1, blue is GTV2,purple is PTV, dark green is CTV1, and dark blue is CTV2. As to the organ at risk, the max total dose of the bilateral temporal lobe (dark green line), the whole brain (black green line), and the brain stem (brown line) were 2.11 Gy, 25.28 Gy, and 22.39 Gy, respectively

The patient received concurrent chemoradiotherapy from October 2021 to December 2021. Radiation regimen: the gross target volume (GTV) encompassed the laryngopharynx and bilateral metastatic neck lymph nodes at 67.84 Gy in 32 fractions (2.42 Gy per traction) over 6–7 weeks, and the total dose for the esophageal tumor was 58.24 Gy in 32 fractions (1.82 Gy per fraction) over 6–7 weeks. The high-risk clinical target volume (CTV) included bilateral cervical lymphatic drainage areas II, III, IV, V, left Ib, and mediastinal lymphatic drainage areas 2, 4, 5, 7 at a dose of 58.24 Gy in 32 fractions (1.82 Gy per fraction). The doses to the organs at risk were as follows: bilateral temporal lobes—2.11 Gy, right temporal lobe—2.11 Gy, and whole brain—25.28 Gy, respectively (Fig. 2). Intravenous cisplatin was administered weekly at a dosage of 40 mg/m^2^ during radiotherapy. The original plan was for 33 fractions of radiotherapy, but the last fraction was cancelled due to persistent hyponatremia (109 mmol/L) of unknown cause after a multidisciplinary discussion. Following this, the patient received two cycles of treatment with Camrelizumab alone (200 mg every 3 weeks).

**Fig. 3:**
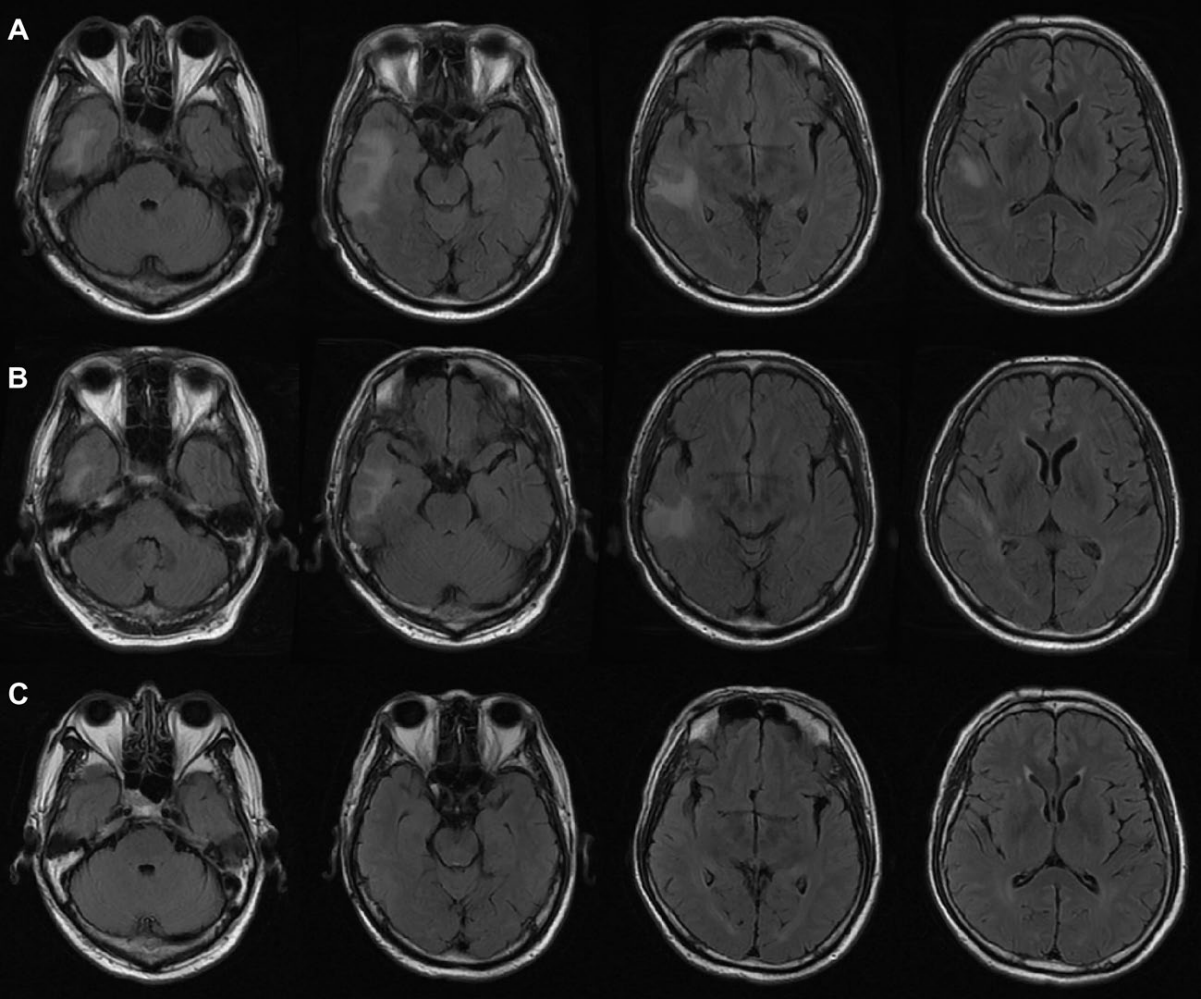
The imaging changes before and after immunosuppressive therapy. **A** Brain MRI showed the first finding of a T2-FLAIR hyperintense signal in the right temporal lobe. **B** Brain MRI showed the focal lesion seemed to diminish after the first 12 days of methylprednisolone, although neurologic symptoms appeared. **C** The latest brain MRI showed that the T2-FLAIR hyperintense signal was resolved

Eight months after receiving the initial dose of Camrelizumab and 3 months after undergoing chemoradiation, the patient presented with symptoms including generalized fatigue, weakness, loss of appetite, and tremors over the past week. The patient was also drowsy but coherent. Serum electrolyte levels showed hyponatremia (128 mmol/L) with low osmolality changes (222.6 mosm/L), while other tests including complete blood count, glucose, liver function, and kidney function were normal. Additional tests were conducted, which revealed a non-significant shift (cortisol 8 am–4 pm–0 am: 0.72–0.53–0.51 µg/dL; ACTH 8 am: 3.327 pg/mL) in serum cortisol and corticotropin levels, indicating no specific findings for hypopituitarism or adrenal insufficiency. Follow-up brain imaging using magnetic resonance imaging (MRI) showed a T2-weighted fluid-attenuated inversion recovery (FLAIR) hyperintense signal in the right temporal lobe (Fig. 3A), though previous positron-emission tomography (PET) scans (Fig. 1C, D) did not show any abnormalities in the corresponding areas. Neurology, imaging, and oncology experts were consulted, and the MRI results were determined to primarily indicate cortical congestion and white matter edema without nodules or masses, ruling out brain metastasis.

The patient underwent a lumbar puncture, which showed normal cerebrospinal fluid (CSF) pressure. CSF analysis revealed increased protein levels (59.64 mg/dL), average glucose levels (3.86 mmol/L), and normal electrolyte levels (potassium 3.04 mmol/L, sodium 142.90 mmol/L, chloride 123.20 mmol/L, and calcium 1.2 mmol/L). Gram staining did not show bacteria, as were mycobacterium tuberculosis and Cryptococcus in the CSF. We also performed CMV and EBV PCR tests on CSF and venous blood due to limited testing conditions. Both tests yielded negative results. Furthermore, IgM antibody tests were performed for HSV1/2, rubella virus, coxsackievirus group B, and adenovirus in the venous blood, and no positive results were obtained. The IgG was 49.50 mg/L (Less than 34 mg/L is customary) in the CSF; but IgM and IgA levels were normal. Additional analysis indicated increased permeability of the blood–brain barrier (7.72 × 10^–3^) (less than 5.0 × 10^–3^ is normal), an elevated IgG index (1.00) (less than 0.85 is standard), and increased IgG-syn levels (13.63 mg/24 h) (less than 7.0 mg/24 h is average). Oligoclonal bands were not detected, and tests for autoimmune encephalitis were negative for relevant antibodies both in the CSF and serum (anti-Recoverin, anti-PKCγ, anti-GAD65, anti-Zic4, anti-Tr, anti-SOX1, anti-Ma2, anti-Ma1, anti-Amphiphysin, anti-CRMP5, anti-Ri, anti-Yo, anti-Hu, anti-NMDA, anti-AMPA1/2, anti-CASPR2, anti-LGI-1, anti-GABAB, anti-DPPX, and anti-mGluR5 antibodies).

**Fig. 4:**
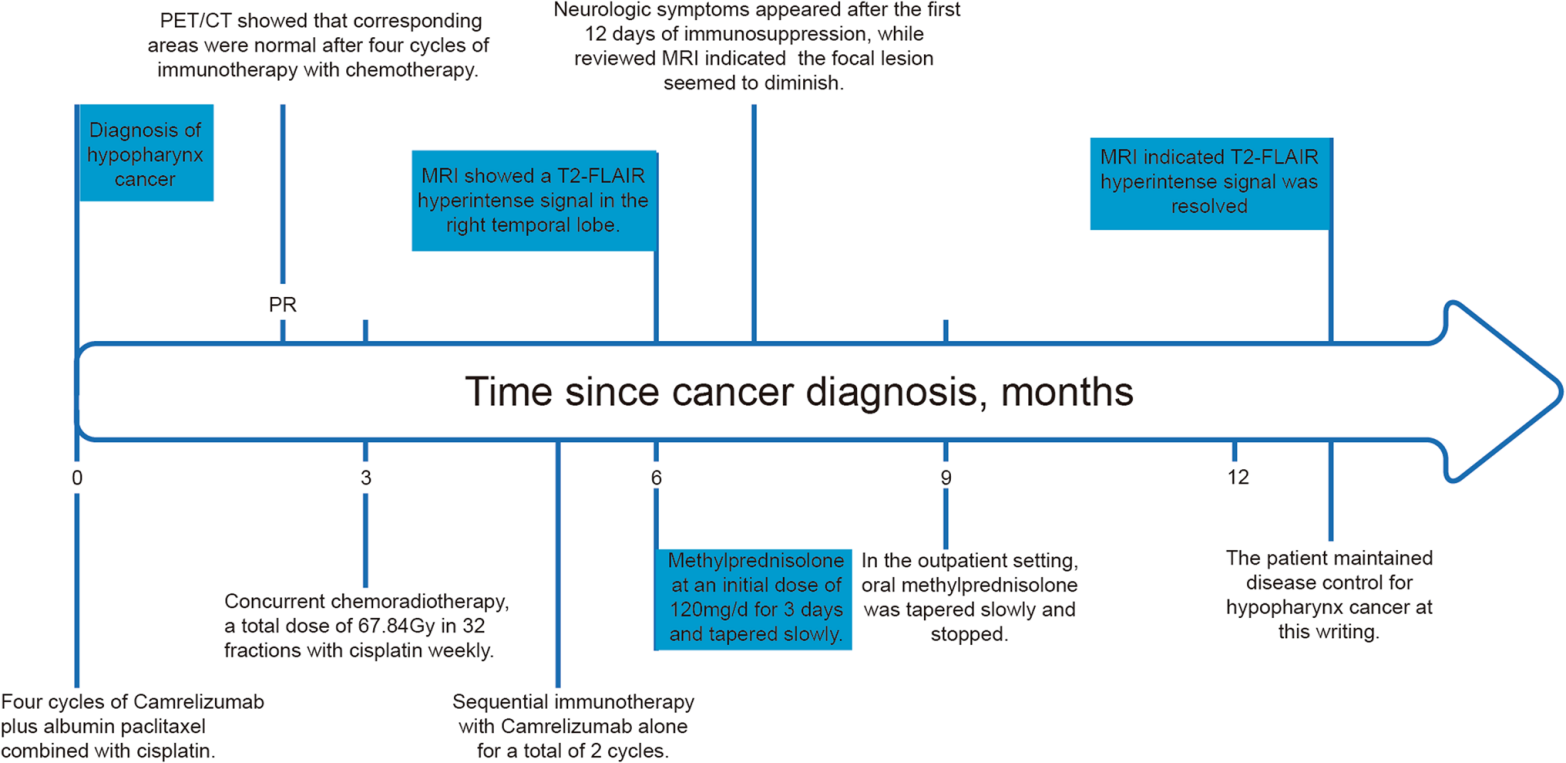
The flowchart of the clinical course for the patient with encephalitis related to Camrelizumab

Considering the suspected autoimmune encephalitis related to immunotherapy, the patient was started on empirical treatment with intravenous methylprednisolone on the sixth day of hospital admission. The initial dose was 2 mg/kg/day for 3 days, followed by a tapering course over 6 weeks (Fig. 4). The patient showed improvement in drowsiness, tremors, and refractory hyponatremia the day after starting immunosuppression treatment, but subsequently developed visual hallucinations attributed to delirium after 12 days of immunosuppression treatment (Fig. 4). A review of the brain MRI suggested that the focal lesion appeared to diminish (Fig. 3B). Olanzapine, 2.5 mg every night, was prescribed to control the new symptoms, leading to an improvement in the patient's condition and discharge from the hospital after 22 days.

In the outpatient setting, the patient underwent a slow tapering of oral methylprednisolone, with no reappearance of neurological symptoms at a dose of 4 mg/day (Fig. 4). Furthermore, 5 months later, the T2-FLAIR hyperintense signal observed on brain imaging (Fig. 3C) resolved completely. The patient experienced a full recovery from NirAE without any neurological sequelae. Additionally, tumor regression was noted even 5 months after discontinuing treatment with Camrelizumab, despite the episode of NirAE.

## Discussion and conclusions

We present a case of encephalitis related to Camrelizumab, a PD-1 inhibitor, in which timely diagnosis and treatment led to a favorable outcome. According to literature, NirAEs have been associated with a negative fallout rate of 23%, including a 7% fatality rate (Ruggiero et al. 2022). The median time from symptom onset to death reported in the literature is 32 days (Wang et al. 2018). Therefore, early recognition of the possibility of NirAEs during treatment is crucial.

When diagnosing immune-related encephalitis, it is important to consider paraneoplastic encephalitis syndrome, which typically precedes a cancer diagnosis and often does not respond well to immunosuppressive therapy. Patients diagnosed with paraneoplastic or immune-related encephalitis may experience complex and overlapping neuropsychiatric symptoms, including memory deficits, cognitive impairments, psychosis, seizures, abnormal movements, and even coma (Dalmau and Graus 2018). Undetected pre-existing paraneoplastic encephalitic syndrome can also be triggered by ICIs and is associated with the poorest prognosis among all types of ICI-induced encephalitis syndromes (Velasco et al. 2021). Paraneoplastic encephalitis syndrome often presents with evidence of inflammation in CSF. A provisional diagnosis of immune-related encephalitis should consider the patient's medical history, clinical features, as well as laboratory and radiological evidence of the central nervous system (CNS). This may include lumbar puncture, serum and CSF antibody testing, and ruling out infections and other potential causes of CNS disorders. A systematic review found that nearly all patients (77 out of 79 [98%]) with immune-related encephalitis displayed abnormalities in their CSF samples, such as pleocytosis or high protein levels. Among these patients, high protein levels (> 0.045 g/dL) were observed in 85% (55 out of 65), with non-focal ICI-induced encephalitis cases having twice the median protein level compared to focal cases (0.15 g/dL) (Velasco et al. 2021). Additional abnormalities found in CSF may include mild to moderate lymphocytic pleocytosis, an elevated IgG index, or the presence of oligoclonal bands (Lawn et al. 2003; Hacohen et al. 2013; Sanchis-Borja et al. 2020). Our patient’s brain MRI revealed a hyperintense signal on T2-weighted FLAIR imaging in the right temporal lobe. The CSF examination revealed a slight elevation in protein and the IgG index, but no abnormalities in autoantibodies were detected. These findings align with reports in the literature, which indicate that the most prevalent laboratory abnormalities seen in cases of ICI-associated encephalitis are bitemporal FLAIR lesions on MRI and monocytic pleocytosis in CSF analysis (Nersesjan et al. 2021). Furthermore, we excluded CSF infection in this patient through gram staining, PCR, and other diagnostic tests. Based on the absence of abnormal lesions in the corresponding areas on previous PET/CT scans, as well as the patient's history of immunotherapy with a PD-1 inhibitor, we concluded that the patient's diagnosis was ICI-induced encephalitis with negative autoantibodies, rather than paraneoplastic encephalitis.

It is important to note that different ICIs may vary in their incidences of immune-related neurotoxicity. After analyzing 59 clinical trials, Cuzzubbo et al. concluded that the overall incidence of NirAEs was 3.8% with anti-CTLA-4 antibodies, 6.1% with anti-PD1 antibodies, and 12.0% with the combination of both. Notably, encephalopathies accounted for 19% of the cases, while meningitis accounted for 15% (Cuzzubbo et al. 2017). A separate meta-analysis supported these findings. It revealed that PD-1/PD-L1 inhibitors were more frequently associated with myasthenia syndromes (50/58, 86%; *P* = 0.005), less common in meningitis (2/13, 15%; *P* < 0.001), and cranial neuropathies (13/31, 42%; *P* = 0.005). On the other hand, CTLA-4 inhibitors were more frequently linked to meningitis (8/13, 62%; *P* < 0.001), and less common in encephalitis (2/56, 4%; *P* = 0.009) and myositis (12/136, 9%; *P* = 0.01). The combination of different ICIs was most frequently associated with cranial neuropathies (12/31, 39%; *P* = 0.005) (Marini et al. 2021). Furthermore, Nivolumab was the drug most frequently implicated, accounting for approximately 57–61% of cases (Stuby et al. 2020; Nersesjan et al. 2021). In the case of our patient, the abnormal lesion observed on MRI was associated with Camrelizumab.

Camrelizumab (AiRuiKa™), a PD-1 inhibitor developed by Jiangsu Hengrui Medicine Co. Ltd, has received conditional approval in People’s Republic of China for the treatment of relapsed or refractory classical Hodgkin lymphoma. Furthermore, it is currently under investigation as a potential treatment for various other malignancies, including B cell lymphoma, esophageal squamous cell carcinoma, gastric/gastroesophageal junction cancer, hepatocellular carcinoma, nasopharyngeal cancer, and non-squamous, non-small cell lung cancer in People’s Republic of China (Lv et al. 2019; Huang et al. 2020; Lan et al. 2020; Qin et al. 2020; Yang et al. 2021a, b; Zhou et al. 2021; Liu et al. 2022; Meng et al. 2022; Ren et al. 2022). However, according to the existing literature, the most common irAEs associated with Camrelizumab are reactive capillary hemangiomas, affecting approximately 58.6% of patients (Li et al. 2020). To date, there have been no documented cases of encephalitis associated with Camrelizumab. Nevertheless, based on the available evidence, our patient potentially represents the first reported case of encephalitis linked to this medication.

There are still several questions that require further attention. Firstly, the literature suggests that the median onset time of NirAEs is 6–8 weeks (Cuzzubbo et al. 2017; Fellner et al. 2018, Stuby et al. 2020; Owen et al. 2021), or these complications may occur after a median of 3–5.5 treatment cycles (Kao et al. 2017; Nersesjan et al. 2021). Interestingly, our patient experienced ICI-associated encephalitis approximately 32 weeks after starting Camrelizumab. Notably, the encephalitis emerged soon after radiotherapy when reviewing the patient's treatment course and the timing of onset (Fig. 4). Thus, we speculate on a potential correlation between radiotherapy and ICI-associated encephalitis. However, the belief that radiation enhances the toxicity of immunotherapy remains controversial. An individual patient data meta-analysis aiming to evaluate if there is an increased risk of serious adverse events (AEs) associated with radiotherapy given within 90 days prior to an ICI enrolled a total of 16,835 patients (Anscher et al. 2022). Ultimately, patients receiving radiotherapy had comparable rates of AEs to those who did not receive radiotherapy. Even in patients with brain metastases who received ICIs combined with radiotherapy, the rates of CNS-related adverse events were not different (8% in ICI combined with radiotherapy; 5% in ICI monotherapy) (Kim et al. 2021). Similarly, a study reported no cases of encephalitis in patients with locally advanced esophageal squamous cell carcinoma who received concurrent chemotherapy and radiotherapy with Camrelizumab (Zhang et al. 2021). However, AEs were more frequent in patients with prior NirAEs when they underwent thoracic radiation (Shaverdian et al. 2020). Nonetheless, there is insufficient evidence to conclude that radiotherapy may have induced encephalitis in this patient. Further research is necessary to precisely explain this result.

Regarding the treatment for ICI-associated encephalitis, Intervention should be initiated promptly upon suspicion of encephalitis, provided that an underlying infectious etiology has been ruled out and no other contraindications are present. Antibody testing results can then be utilized to refine or alter the treatment plan (Graus et al. 2016; Abboud et al. 2021). Currently, a standard treatment for autoimmune encephalitis is lacking, but immunosuppressive therapy with high-dose corticosteroids or intravenous immunoglobulin is considered a first-line therapy. Additionally, methylprednisolone can be used at a dose of 1–2 mg/kg/day for treating encephalitis. In the presence of severe or progressing symptoms, corticosteroids (methylprednisolone 1 g IV daily for 3–5 days) in combination with intravenous immune globulin (IVIG) at a dose of 2 g/kg over 5 days or plasmapheresis. Taper steroids gradually after acute management is completed, for a duration of at least 4–6 weeks. If positive for autoimmune encephalopathy, paraneoplastic antibody, or limited or no improvement, consider rituximab in consultation. Continuous vigilance is necessary since ICI-associated encephalitis is susceptible to relapse despite receiving steroid therapy (Taillefer et al. 2020). In our report, the patient was administered intravenous methylprednisolone at a daily dose of 120 mg for 3 days, and underwent a tapering course of corticosteroids over 6 weeks. Although the patient's neurological symptoms had transiently worsened, we reviewed MRI results and continued tapering corticosteroids due to improved imaging findings. Therefore, close monitoring of the patient's condition and periodic review of cranial MRI are necessary during treatment. Finally, the patient exhibited complete recovery with no abnormal imaging findings. Overall, literature data suggest that early treatment is crucial in reducing the morbidity associated with NirAEs (Touat et al. 2017). Failure to provide proper treatment for any NirAE may lead to treatment termination, failure, and potentially life-threatening outcomes (Chen et al. 2015).

In conclusion, while no previous cases related to Camrelizumab have been reported, it is essential to remain vigilant and consider irAEs triggered by ICIs as potential diagnoses for newly occurring neurologic syndromes or unexplained intracranial lesions. The use of ICIs in patients with malignant tumors is becoming increasingly common. Early identification and proper management of NirAEs are crucial for enhancing patient outcomes and facilitating treatment planning. Additionally, further studies are warranted to explore strategies for rapid diagnosis and optimal treatment of ICI-associated encephalitis.

## Data Availability

All data generated or analyzed during this study are included in this published article.
